# Advanced Glycation End-Products Induce Apoptosis of Vascular Smooth Muscle Cells: A Mechanism for Vascular Calcification

**DOI:** 10.3390/ijms17091567

**Published:** 2016-09-16

**Authors:** Sayo Koike, Shozo Yano, Sayuri Tanaka, Abdullah M. Sheikh, Atsushi Nagai, Toshitsugu Sugimoto

**Affiliations:** 1 Department of Internal Medicine 1, Shimane University Faculty of Medicine, Shimane 693-8501, Japan; syoko@med.shimane-u.ac.jp (S.K.); s-tanaka@med.shimane-u.ac.jp (S.T.); sugimoto@med.shimane-u.ac.jp (T.S.); 2 Department of Laboratory Medicine, Shimane University Faculty of Medicine, Shimane 693-8501, Japan; abdullah@med.shimane-u.ac.jp (A.M.S.); anagai@med.shimane-u.ac.jp (A.N.)

**Keywords:** advanced glycation end-products, apoptosis, vascular smooth muscle cell, NAD(P)H oxidase, oxidative stress, vascular calcification, diabetes, chronic kidney disease

## Abstract

Vascular calcification, especially medial artery calcification, is associated with cardiovascular death in patients with diabetes mellitus and chronic kidney disease (CKD). To determine the underlying mechanism of vascular calcification, we have demonstrated in our previous report that advanced glycation end-products (AGEs) stimulated calcium deposition in vascular smooth muscle cells (VSMCs) through excessive oxidative stress and phenotypic transition into osteoblastic cells. Since AGEs can induce apoptosis, in this study we investigated its role on VSMC apoptosis, focusing mainly on the underlying mechanisms. A rat VSMC line (A7r5) was cultured, and treated with glycolaldehyde-derived AGE-bovine serum albumin (AGE3-BSA). Apoptotic cells were identified by Terminal deoxynucleotidyl transferase dUTP nick end labeling (TUNEL) staining. To quantify apoptosis, an enzyme-linked immunosorbent assay (ELISA) for histone-complexed DNA fragments was employed. Real-time PCR was performed to determine the mRNA levels. Treatment of A7r5 cells with AGE3-BSA from 100 µg/mL concentration markedly increased apoptosis, which was suppressed by Nox inhibitors. AGE3-BSA significantly increased the mRNA expression of NAD(P)H oxidase components including Nox4 and p22^phox^, and these findings were confirmed by protein levels using immunofluorescence. Dihydroethidisum assay showed that compared with cBSA, AGE3-BSA increased reactive oxygen species level in A7r5 cells. Furthermore, AGE3-induced apoptosis was significantly inhibited by siRNA-mediated knockdown of Nox4 or p22^phox^. Double knockdown of Nox4 and p22^phox^ showed a similar inhibitory effect on apoptosis as single gene silencing. Thus, our results demonstrated that NAD(P)H oxidase-derived oxidative stress are involved in AGEs-induced apoptosis of VSMCs. These findings might be important to understand the pathogenesis of vascular calcification in diabetes and CKD.

## 1. Introduction

Vascular complication is an important aspect of the pathological course of diabetes mellitus, and affects the disease-related morbidity and mortality. For the development of such complications, hyperglycemia is suggested to play a central role. Hence, a long term intensive control of glycemic status is demonstrated to play a pivotal role in the prevention of the complication [1,2,3]. For example, the Diabetes Control and Complications Trial/Epidemiology of Diabetes Interventions and Complications (DCCT/EDIC) Study revealed that an intensive diabetes management of a mean of 6.5 years decreased the incidence of cardiovascular (CV) disease in type 1 diabetes [1]. Similarly, United Kingdom Prospective Diabetes Study (UKPDS) demonstrated that intensive glycemic control started at the time of diagnosis is associated with a significantly reduced risk of microvascular disease, myocardial infarction and death from any cause in type 2 diabetes [2]. Such findings suggest a salutary effect of intensive therapy on the risk of CV events. A significantly improved glycemic control was seen in intensive therapy mainly during early phage of the treatment, and such difference usually lost afterwards. However, its beneficial effects persist even when the glycemic control was similar to conventional therapy. The reason of such lasting effect of intensive glycemic control is not very clear. Nevertheless, it is proposed that hyperglycemia-induced early metabolic disturbance in conventional therapy could exert a “legacy” effect on diabetic vessels, which persists even in good glycemic control afterwards. Among the metabolic system implicated in diabetic vascular complications, the properties of advanced glycation end-products (AGEs) well explains the “legacy” effect owing to its difficulty in degradation and clearance from the diabetic tissue [3].

During generation of AGE, reducing sugars including glucose first react non-enzymatically with proteins in Maillard reaction. After further rearrangement reactions and cross linking, these products of Maillard reaction ultimately generate AGEs. AGEs are generated not only by glucose but also by dicarbonyl (glyoxal, methylglyoxal, and 3-deoxyglucoson) or hydroxylaldehyde (glycelaldehyde and glycolaldehyde) compounds. In addition to diabetic patients, blood concentration of AGEs showed markedly high in patients undergoing chronic dialysis, suggesting them to be uremic toxins that accumulate in end-stage chronic kidney disease (CKD) [4]. Accumulation of AGEs has known to be related to the pathology of various disorders including arteriosclerosis, myocardial hypertrophy, chronic inflammation and dialysis amyloidosis. In these conditions, AGE can be a predictor of CV events and mortality [5,6,7]. Several receptors for AGEs have been identified. Among them receptor for AGE (RAGE) is important, because of its ability to activate various signaling pathways that lead to diverse downstream effects. Nuclear factor (NF)-κB sites and interferon-γ response element are located on RAGE promoter, linking RAGE to inflammation. Moreover, AGE-RAGE signaling can induce oxidative stress and tumor necrosis factor–α, both of them activate NF-κB, thus form a positive feed-back loop leading to the development of CV diseases [8]. Such findings are supported by study with diabetes model mouse, showing that the defect of RAGE prevented the development of AGEs-induced atherosclerosis [9].

Möenckeberg-type arterial calcification in the media is a characteristic feature of diabetic, as well as CKD patients that develops and progresses time dependently. The severity of such medial calcification has been known to be a predictor of CV events and mortality [10,11]. As a mechanism of vascular calcification, vascular smooth muscle cells (VSMCs) apoptosis is reported to be important. Previous studies have reported that AGEs accelerate vascular calcification in a RAGE-dependent manner [12,13]. Moreover, AGEs suppressed cellular differentiation and maturation, and induced apoptosis in many type of cells including retinal microvascular endothelium cells, retinal epithelial cells, neuronal cells, osteoblast precursor cells, and osteocytes [14,15,16,17,18]. However, the effects of AGEs on VSMC apoptosis, and its relation with vascular calcification are largely unknown. Previously, we have demonstrated that glycolaldehyde-derived AGE induces calcium deposition in rat VSMCs through excessive generation of reactive oxygen species (ROS) and phenotypic transition into osteoblast like cells [19]. In this study, we aimed to investigate the role of glycolaldehyde-derived AGE on VSMC apoptosis, with emphasis on the underlying mechanism. We found that AGE-induced ROS generation via NAD(P)H oxidase plays an important role in VSMC apoptosis, as it does in calcium deposition [19].

## 2. Results

### 2.1. AGE3-BSA Increased Calcium Deposition in Vascular Smooth Muscle Cells (VSMCs)

**Figure 1:**
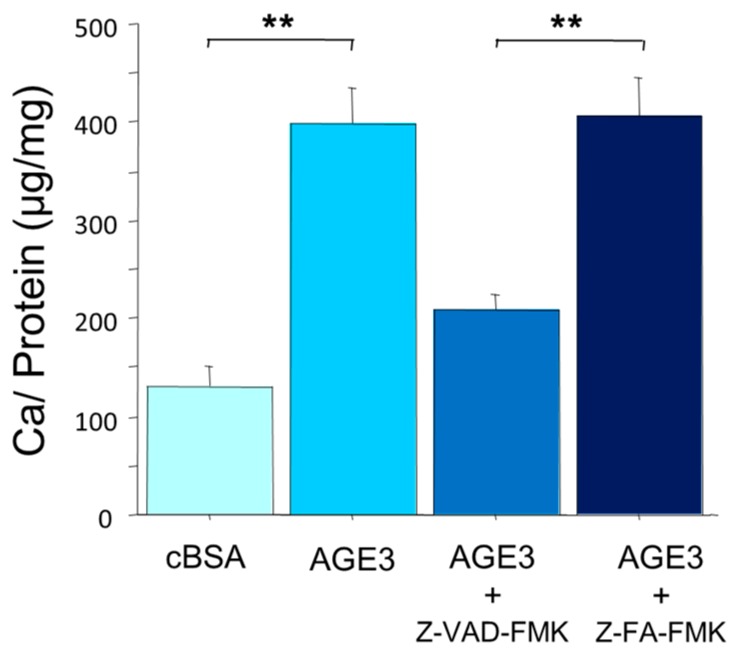
Glycolaldehyde-derived advanced glycation end-products-bovine serum albumin (AGE3-BSA) (100 µg/mL) increased calcium deposition in a rat vascular smooth muscle cell line and it was inhibited by caspase inhibitor. After reaching confluency, A7r5 cells were incubated with calcification medium containing control BSA (cBSA) or AGE3-BSA in the presence or absence of general caspase inhibitor Z-VAD-FMK (10 µM) or the control Z-FA-FMK (10 µM) for three days. Then, the calcium deposition was measured as described in the Method Section. To determine statistical significance, the results were analyzed by unpaired *t*-test, and the statistical significance was denoted as follows, ** *p <* 0.001.

A7r5 cells were cultured in a growth medium till reaching confluency. Then, the cells were treated with control BSA (cBSA) or AGE3-BSA in calcification medium. After incubation for three days, calcium content was measured by the *O*-cresolphthaleincomplexone, and the results were normalized by total protein content. This method is superior to the quantification of the degree of calcium deposition, and suitable for determining late phase of calcium deposition, whereas microscopic imaging method is suitable for the detection of calciprotein particles, which are participated in an early phase of mineralization. The results showed that calcium deposition was significantly increased by AGE3-BSA (131 vs. 399 for cBSA and AGE3-BSA, respectively; *p <* 0.001) (Figure 1).

To examine effects of apoptosis on calcium deposition, A7r5 cells were treated with general caspase inhibitor Z-VAD-FMK (10 µM) or the control Z-FA-FMK (10 µM) for three days. AGE3-BSA -induced calcium deposition was significantly inhibited by the treatment with caspase inhibitor (208 vs. 407 for Z-VAD-FMK and Z-FA-FMK, respectively; *p <* 0.001) (Figure 1). This suggests that AGE-induced calcium deposition is mediated by apoptotic cell death in VSMCs. Thus, we investigate AGE-induced apoptosis and the mechanism in A7r5 cells.

### 2.2. AGE3-BSA Induced Apoptosis of VSMCs

**Figure 2:**
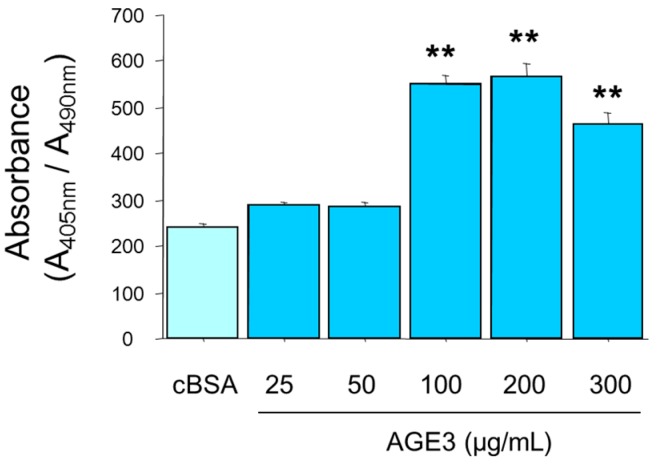
AGE3-BSA treatment induced apoptosis in A7r5 rat vascular smooth muscle cells. The cells were treated with cBSA, or indicated concentrations of AGE3-BSA, and the levels of apoptotic cells were measured using an ELISA-based method, as described in the Method section. Apoptosis was found to be increased by AGE3-BSA after treatment for five days. The results are presented here as averages ± SE of at least three independent experiments. The statistical significance of the results was analyzed by one-way ANOVA followed by LDS post-hoc test. Statistical significance was denoted as follows, ** *p <* 0.001 vs. cBSA.

A7r5 cells were cultured in growth medium until confluency. Then the cells were treated with cBSA, or increasing concentration of AGE3-BSA (25, 50, 100, 200, and 300 µg/mL). Calcification medium was changed twice a week. On Day 3 and 5, apoptotic cell death was measured using an ELISA-based method. The results showed that up to 50 µg/mL concentration, AGE3-BSA did not affect A7r5 apoptosis (240 vs. 289 and 284 for cBSA, 25 µg/mL and 50 µg/mL of AGE3-BSA, respectively; not significant). AGE3 significantly increased apoptosis from 100 µg/mL concentration (Figure 2). However, we did not find any dose-dependent effect of AGE3-BSA beyond 100 µg/mL concentration (551, 556, and 463 for 100, 200 and 300 µg/mL of AGE3-BSA, respectively) (Figure 2).

### 2.3. AGE3 Induced VSMC Apoptosis through NAD(P)H Oxidase Activity

**Figure 3:**
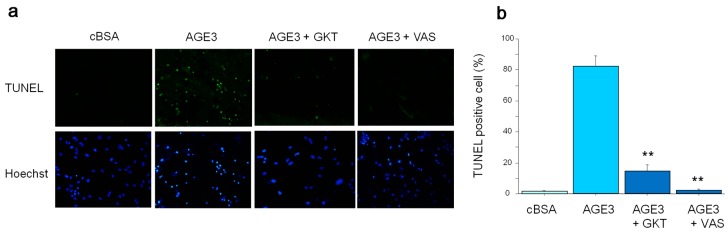
AGE3-BSA-induced apoptosis in A7r5 cells was mediated by NAD(P)H oxidase. (**a**) Cultured A7r5 cells were incubated in calcification medium containing cBSA, or AGE3-BSA (100 µg/mL) in the presence or absence of NAD(P)H oxidase inhibitors including GKT137831 (20 µM) or VAS2870 (10 µM) for three days. Apoptosis was evaluated by TUNEL assay, as described in the Method section. Cells in the culture were identified by nuclear staining with Hoechst, and evaluated under a fluorescence microscope; (**b**) For quantification, Hoechst and TUNEL double positive cells were counted in 10 random microscopic fields at 200× magnification, and expressed as percent TUNEL positive cells in a culture. Statistical significance of the results was analyzed by one-way ANOVA followed by LDS post-hoc test. Statistical significance was denoted as follows, ** *p <* 0.001 vs. AGE3-BSA.

As AGE3-BSA showed maximum apoptotic effect at 100 µg/mL concentration, in all subsequent experiments, we used this dose to investigate about the underlying mechanism of apoptosis. To examine further about apoptosis, cultured A7r5 cells were incubated with cBSA or AGE3-BSA (100 µg/mL) for three days. After treatment, analysis of apoptosis by TUNEL assay showed that AGE3-BSA markedly increased TUNEL positive cells (Figure 3a). Interestingly, pretreatment of cells with NAD(P)H oxidase inhibitor including GKT137831 (20 µM) or VAS2870 (10 µM), markedly decreased the number of TUNEL positive cells (Figure 3a). Quantification analysis also showed that the percentage of TUNEL positive cells in a total cell culture population was significantly increased by AGE3-BSA treatment (1% vs. 83% for cBSA and AGE3-BSA, respectively; *p <* 0.001), and such effect of AGE3-BSA was greatly inhibited by NAD(P)H oxidase inhibitors (14% and 2% for GKT137831 and VAS2870, respectively) (Figure 3b). These findings suggest that AGE3-BSA-induced apoptosis of VSMC was mediated by the activation of NAD(P)H oxidase.

### 2.4. AGE3-BSA Induced Expression of NAD(P)H Oxidase Components and ROS Generation in VSMCs

**Figure 4:**
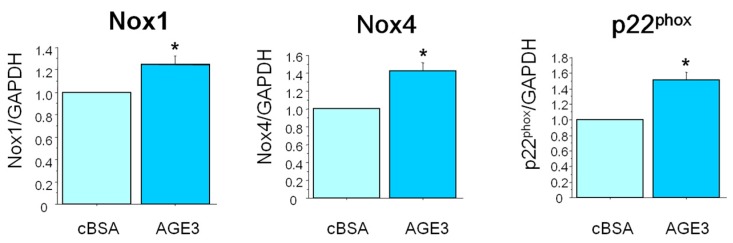
AGE3-BSA increased the mRNA expression of the components of NAD(P)H oxidase. A7r5 cells were treated with cBSA or AGE3-BSA (100 µg/mL) for three days. Total mRNA was isolated, and Nox1, Nox4 and p22^phox^ mRNA levels were evaluated by real-time PCR, as described in the Method section. GAPDH mRNA was used as a loading control. The data presented here as averages ± SE of at least three experiments. The statistical significance of the results was analyzed by unpaired *t*-test. The statistical significance was denoted as follows, * *p <* 0.05 vs. cBSA.

To evaluate further about the roles of NAD(P)H oxidase in AGE-induced apoptosis of VSMC, we checked the effects of AGE3-BSA on the mRNA expression of the components of NAD(P)H in A7r5 cells. Three days after incubation with 100 µg/mL of AGE3-BSA or cBSA, total RNA was isolated from A7r5 cells, and the mRNA expression of Nox1, Nox4 and p22^phox^ was assessed by real-time PCR. The results showed that AGE3-BSA (100 µg/mL) treatment significantly increased the expression of Nox1, Nox4 and p22^phox^ mRNA (24%; *p <* 0.05, 43%; *p <* 0.05, and 51%; *p <* 0.05, respectively) (Figure 4).

**Figure 5:**
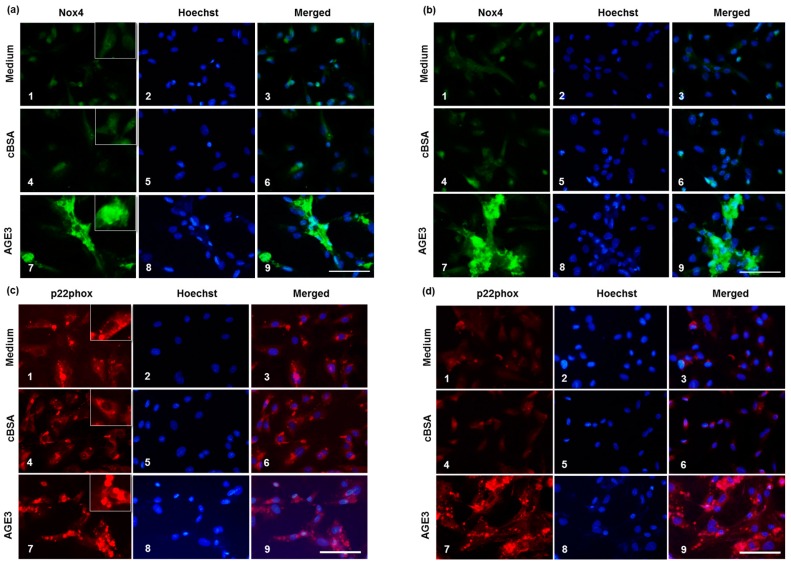

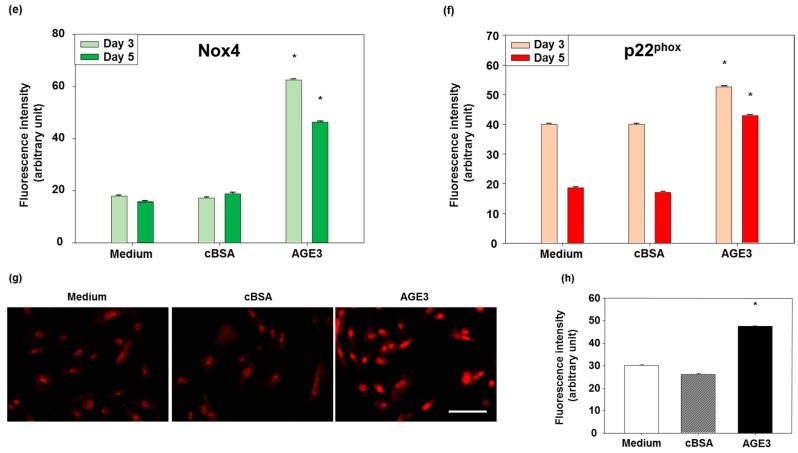
Effects of AGE3 on the protein expression of Nox4 and p22^phox^. A7r5 cells were treated with medium alone, cBSA or AGE3-BSA for three and five days. After treatment, Nox4 and p22^phox^ proteins were analyzed by immunofluorescence staining, as described in Materials and Method. Representative photomicrographs of Nox4 immunofluorescence staining of Day 3 and 5 are shown in (**a**,**b**) respectively, and quantified fluorescence intensities in (**e**). 1–3; medium only (no treatment), 4–6; cBSA treatment, and 7–9; AGE3 treatment. Representative photomicrographs of p22^phox^ immunofluorescence staining of Day 3 and 5 are shown in (**c**,**d**) respectively, and quantified fluorescence intensities in (**f**). Higher magnification photomicrographs are shown in the insets for localization of the expressed proteins. Cellular ROS levels were analyzed by DHE assay after treating the cells for five days. Representative photomicrographs of DHE fluorescence are shown in (**g**); and quantified fluorescence intensities in (**h**). Numerical data are presented here as average ± SE of at least four experiments. * *p <* 0.05 vs. medium or cBSA of same time period. Scale bar = 100 µm.

We further analyzed the expression of Nox4 and p22^phox^ at the protein level. Immunofluorescence results demonstrated that Nox4 protein level was barely detectable in medium treated cells both at Day 3 and 5, whereas p22^phox^ level was quite high at Day 3, which was decreased at Day 5. AGE3-BSA treatment increased Nox4 protein both at Day 3 and 5 (Figure 5a,b,e). In the case of p22^phox^, the protein level was increased slightly, but significantly by AGE3-BSA at Day 3 comparing medium treated and cBSA treated cells (Figure 5c,f). However, AGE3-BSA considerably increased p22^phox^ protein level at Day 5 (Figure 5d,f).

Next, we checked whether such increased production of Nox4 and p22^phox^ proteins by AGE3-BSA has any functional significance. A7r5 cells were treated with medium, cBSA or AGE3-BSA, and cellular ROS levels were checked by dihydroethidium (DHE) assay. The results showed that AGE3-BSA significantly increased cellular ROS level compared to medium treated and cBSA treated conditions (Figure 5g,h).

### 2.5. Silencing Nox4 and p22^phox^ Suppressed AGE3-Induced Apoptosis of A7r5 Cells

**Figure 6:**
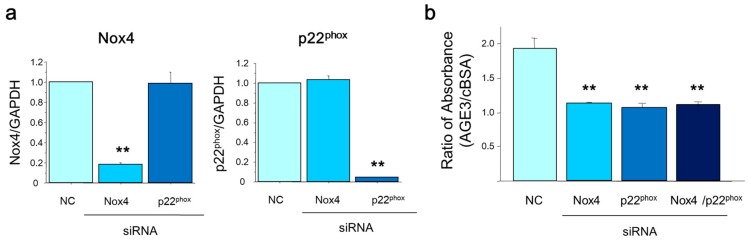
AGE3-BSA-induced apoptosis is mediated through Nox4 or p22^phox^. (**a**) Nox4 or p22^phox^ siRNA was transfected to A7r5 cells, total RNA was isolated three days after transfection, and Nox4 and p22^phox^ mRNA levels were measured by real-time PCR, as described in the Method section. GAPDH mRNA was used as loading control. A scramble siRNA was used as a negative control (NC); (**b**) AGE3-BSA-induced apoptosis was inhibited by silencing of Nox4 and p22^phox^ mRNA. Apoptosis was evaluated by cell death detection ELISA kit. The absorbance in cells treated with AGE3-BSA was compared with that in cells treated with cBSA. The ratio was demonstrated after correction with each cBSA. Results were analyzed by one-way ANOVA and LDS post-hoc test. ** *p <* 0.001 vs. NC.

Next, we examined effects of the silencing of Nox4 and p22^phox^ on AGE-induced apoptosis using mRNA-specific siRNA transfection in A7r5 cells. The real time PCR results showed that both Nox4 and p22^phox^ mRNA levels were decreased to 5%–20% after mRNA specific siRNA transfection, indicating their sufficient silencing effect (Figure 6a). Importantly, AGE3-BSA-induced A7r5 apoptosis was markedly inhibited (42% or 47%) by transfection of either Nox4 or p22^phox^ siRNA, compared to control (scramble siRNA transfection) (Figure 6b). However, we did not find any synergistic or additive effect of double silencing of Nox4 and p22^phox^ on the apoptosis (42% inhibition; not significant vs. Nox4 siRNA or p22^phox^ siRNA transfection).

## 3. Discussion

We have previously shown that AGEs stimulate calcium deposition in VSMCs through excessive ROS generation and phenotypic transition to osteoblastic cells [19]. In the present study, we observed that AGE3-BSA significantly induced calcium deposition and apoptosis in A7r5 cells. The mRNA and protein expression of Nox4 and p22^phox^ was up-regulated by AGE3-BSA treatment. Moreover, AGE3-induced apoptosis was significantly suppressed by pretreating the cells with Nox inhibitors. Nox4 and p22^phox^ silencing showed similar inhibitory effects on the apoptosis. Taken together, AGE3-induced apoptosis of VSMCs might be the result of NAD(P)H oxidase activation and ROS generation.

In vasculature, ROS is generated mainly by NAD(P)H oxidase. In a previous study, oxidized LDL causes NAD(P)H oxidase mediated excessive ROS generation, resulting accelerated apoptosis of VSMCs [20]. Diabetic model mice showed elevated expression of Nox, bone morphogenetic protein-4, and osteopontin (OPN) genes in the aorta, which was suppressed by TEMPOL, a superoxide scavenger [21]. Patients with type 1 diabetes showed a positive association between the concentration of pentosidine, one of AGEs, and 8-OHdG in the urine [22]. We have previously observed that 8-OHdG level in the culture medium was elevated by AGE3 treatment in VSMCs, indicating that AGE stimulates ROS production [19]. Since excessive ROS is thought to stimulate the production of AGEs [23,24], the generation of AGEs and ROS might activate a positive feedback loop.

NAD(P)H oxidase is composed of Nox isoforms, p22^phox^, and associated proteins such as p47^phox^ as a subunit. According to recent studies, Nox1, Nox4, p22^phox^ and p47^phox^ are expressed in VSMCs, and Nox4 and p22^phox^ possess significant functionality among them [25,26,27,28,29,30]. Using gene targeting mouse model, their functionality in the vessel has been proved; p22^phox^ is involved in the progression of atheroma [31], while Nox4 facilitates cardiac adaptation to chronic stress such as pressure overload or hypoxia [32]. In addition, Nox4 plays a key role in the pathogenesis of diabetic nephropathy by targeting renal fumarate hydratase, the enzyme that increases fumarate levels [33]. In the present study, AGE3-induced apoptosis was suppressed by silencing Nox4 or p22^phox^, and double silencing did not show any synergistic, or even additive effects, indicating that the presence of both Nox4 and p22^phox^ is essential for functional activity of NAD(P)H oxidase. Moreover, the function of one of them cannot compensate the other components. As previous studies demonstrated that the activity of p22^phox^ as well as Nox4 is associated with their mRNA levels [34,35], increased expression of Nox4 or p22^phox^ is most probably responsible for ROS generation. Since silencing of Nox1 did not affect AGE-induced calcium deposition in our previous study [19], we speculate that expression level of Nox1 is of little importance for calcium deposition in VSMCs. Future studies need to address the roles of Nox1 and p47^phox^ in VSMCs.

According to previous works, various mechanisms such as growth arrest specific gene 6 (*gas6*) mediated Axl-PI3kinase-Akt pathway, osteoprotegerin/RANKL-RANK system, activation of matrix metalloproteinases, inflammatory cytokines, and inhibiting factors including pyrophosphate, matrix Gla protein, and α2-HS-glycoprotein (fetuin-A) are involved in the development of vascular calcification [36,37,38,39]. Regarding signal transduction, Tanikawa and coworkers indicated that AGEs-induced VSMC calcification is mediated through RAGE and p38 MAPK pathway [40]. Since activation of p38 MAPK is associated with excessive ROS production and cellular apoptosis [41], there is a possibility that AGE-induced apoptosis promotes vascular calcification through the RAGE/p38 MAPK pathway. On the other hand, high concentration of extracellular phosphate induced apoptosis and calcium deposition in VSMCs through Gas6-Axl interaction [36]. It was demonstrated that atorvastatin inhibited calcification by preventing apoptosis without affecting mevalonate pathway. Although the molecular mechanisms of apoptosis and vascular calcification may differ between AGEs and phosphate, further study is necessary to elucidate the mechanisms involved in the pathogenesis of AGEs-induced or ROS-mediated calcification of VSMCs.

In medial calcification, loss of VSMCs has been observed. In uremic model mice, VSMC phenotype change and VSMC loss was observed earlier than the progression of calcification [42]. In the aorta of CKD patients, apoptotic cell death was observed in vascular calcified area [43]. In diabetes or hyperglycemic condition, VSMCs exhibited significantly increased rates of proliferation, apoptosis, and migration, and decreased expression of contractile regulating proteins as well as abnormal cellular morphology, suggesting that normal vascular structure and function are impaired [44,45,46,47,48]. Therefore, prevention of AGE generation or AGE-RAGE interaction can be a therapeutic target of the progression of arteriosclerosis including vascular calcification [13]. In addition, a very recent study has shown that AGE-induced VSMC apoptosis and ER stress are augmented by treatment with a novel carboxymethylated peptide [46].

## 4. Materials and Methods

### 4.1. Cell Culture

A7r5 cell, a rat aortic VSMC line, was obtained from European Collection of Cell Cultures through Dainippon Seiyaku (Osaka, Japan). The cells were cultured in fully humidified atmospheric air containing 5% CO_2_ condition at 37 °C temperature, with Dulbecco’s modified Eagle’s medium (DMEM) supplemented with 10% FBS, 100 U/mL penicillin and 100 U/mL streptomycin.

### 4.2. Induction of Calcification

Calcification of A7r5 was induced following the method described by Shioi et al. [49]. Briefly, after reaching confluency, the growth medium of A7r5 was changed to calcification medium (DMEM containing 10% FBS, 10 mM sodium pyruvate, 10^−7^ M insulin, 100 U/mL penicillin, 100 mg/mL streptomycin, and β-glycerophosphate). The medium was replaced with fresh medium twice a week. Treatment with general caspase inhibitor Z-VAD-FMK (Abcam, Cambridge, UK) or the control Z-FA-FMK (Abcam) was performed to examine an effect of apoptosis on calcium deposition.

### 4.3. Preparation of AGEs

AGE-BSA was prepared as described previously [50]. Briefly, BSA was incubated under sterile conditions with glycolaldehyde (AGE3) (Sigma Aldrich, St. Louis, MO, USA) and 5 mM diethylenetriamine pentaacetic acid (DTPA) in 0.2 M phosphate buffer (pH 7.4) at 37 °C for seven days. Then, the low molecular weight reactants and aldehydes were removed using a PD-10 chromatography column and dialysis against PBS.

### 4.4. Quantification of Calcium Deposition

Cells were decalcified with 0.6 N HCl for 24 h. The calcium content of HCl supernatant was determined colorimetrically by *o*-cresolphthaleincomplexone method (calcium *C*-test Wako; Wako Pure Chemical Industries, Osaka, Japan). After decalcification, cells were washed three times with PBS and solubilized with 0.1 N NaOH/0.1% SDS. Protein content was measured with the Pierce BCA Protein Assay kit (Thermo Fisher Scientific, Waltham, MA, USA). Calcium content of the cell layer was normalized by protein content.

### 4.5. TUNEL Assay

Terminal deoxynucleotidyl transferase dUTP nick end labeling (TUNEL) was done using a kit according to the manufacturer’s protocol (In Situ Cell Death Detection Kit, POD, Roche Molecular Biochemicals, Mannheim, Germany). Briefly, A7r5 cells were seeded on a chamber slide at a density of 2 × 10^4^ cells/well and cultured overnight in DMEM with 10% FBS and antibiotics. On the next day, the cells were treated with AGE3 or control BSA in the presence or absence of NAD(P)H oxidase (Nox) inhibitors, GKT137831 (Selleck Chemicals, Houston, TX, USA) or VAS2870 (Sigma Aldrich) for three days. After fixation with 4% paraformaldehyde and permeabilized with 0.1% Triton X-100, the DNA nicks in apoptotic cells were labeled with fluorescein-conjugated nucleotides using labeling solutions provided by the manufacturer. To identify cells, nuclei were stained with Hoechst. Apoptosis was semi-quantitatively evaluated by the ratio of TUNEL positive cell number divided by Hoechst positive cell number. Briefly, 10 fields were randomly selected in each staining of each slide, and the positive cell number was counted by two different observers under the fluorescence microscope at the same condition to calculate the ratio.

### 4.6. Apoptosis Assay Using a DNA Fragment Detection ELISA Kit

A7r5 cells were seeded on a 96-well plate at a density of 2 × 10^4^ cells/well, and cultured overnight in DMEM with 10% FBS and antibiotics. On the next day, the cells were treated with AGE3 or control BSA (cBSA). After five days, the cells were lysed and the supernatant was analyzed for DNA fragments using an enzyme-linked immunosorbent assay (ELISA) kit, according to the manufacturer’s protocol (Cell Death Detection ELISA, Roche Molecular Biochemicals).

### 4.7. Quantification of mRNA Expression by Real-Time PCR

Total RNA was isolated from cultured A7r5 cells using Trisol reagent (Invitrogen, San Diego, CA, USA) according to the manufacturer’s recommended protocol. First-strand cDNA was synthesized using oligo-dT primer and SuperScript III cDNA synthesis kit (Invitrogen). SYBR green chemistry was used to perform quantitative determination of the mRNAs, following an optimized protocol [51]. The design of sense and antisense oligonucleotide primers was based on published cDNA sequences using the Primer Express software (version 2.0.0, Applied Biosystems, Carlsbad, CA, USA). The cDNA was amplified using an ABI PRISM 7000 sequence detection system (Applied Biosystems). The cDNA-specific SYBR Green Mix was incorporated into the PCR buffer provided in the QuantiTect SYBR PCR kit (QIAGEN, Valencia, CA, USA) to allow for quantitative detection of the PCR product. The temperature profile of the reaction was 60 °C for 2 min, followed by 95 °C for 15 min, and 40 cycles of denaturation at 94 °C for 15 s, and annealing and extension at 60 °C for 1 min.

### 4.8. RNA Interference

RNA interference was used to down-regulate the expression of Nox4 and p22^phox^ in A7r5 cells. SMARTpool small interfering RNA (siRNA) and SMARTpool reagents for these genes, and nonspecific control siRNA duplexes were designed and synthesized by Customer SMARTpool siRNA Design from Dharmacon (Lafayette, CO, USA). For gene knock down experiments, A7r5 cells were plated in 1.5 cm dish and cultured for 24 h in DMEM containing 10% FBS and antibiotics. Next, after 24 h incubation in medium without antibiotics, cells were transfected with siRNAs (25 nM) using transfection reagent according to the manufacturer’s instructions. After another 48 h of culture, cells were incubated in calcification medium.

### 4.9. Immunofluorescence

A7r5 cells were cultured in a Lab-Tek II Chamber Slide (Thermo Fisher Scientific Inc., Yokohama, Japan). After appropriate treatment, the medium was removed, cells were washed once with PBS, and fixed with 100% methanol. After blocking with a solution containing 0.1% Triton-X100, and normal goat of horse serum, the cells were incubated with anti-NOX4 IgG (goat, 1:200, Santa Cruz, Dallas, TX, USA), or anti-p22^phox^ IgG (rabbit, 1:200, Santa Cruz) for 2 h at room temperature. FITC conjugated anti-goat IgG, and Texas red conjugated anti-rabbit IgG were used to detect Nox4 and p22^phox^, respectively. To identify the cells, nuclei were visualized with Hoechst staining. Then stained cells were examined under a fluorescence microscope, and photographs were taken. The fluorescence intensities were quantified using ImageJ software (http://imagej.net/ImageJ).

### 4.10. DHE Measurement

To determine cellular ROS levels, DHE assay was done following a previously described method [52]. Briefly, after treatment, cells were washed once with PBS, and incubated in serum free medium containing 30 µM DHE for 3 min at room temperature on a rocking platform after protecting from light. Then the cells were washed once with PBS and quickly examined under a fluorescence microscope and photographs were taken. The DHE fluorescence intensities were quantified using ImageJ software.

### 4.11. Statistics

The numerical data are expressed as mean ± SEM. Statistical evaluation of the differences between the groups was carried out with unpaired *t*-test and/or one-way analysis of variance (ANOVA) followed by Fisher’s protected least significant difference (LSD). For all statistical tests, a value of *p <* 0.05 was considered to be statistically significant difference.

## 5. Conclusions

AGEs stimulate VSMC apoptosis through excessive ROS generation. Component of NAD(P)H oxidase such as Nox4 may be a good candidate of new strategy to prevent vascular calcification.

## Abbreviations

AGEs	Advanced glycation end-products
BSA	Bovine serum albumin
CKD	Chronic kidney disease
CV	Cardiovascular
ELISA	Enzyme-linked immunosorbent assay
NADPH	Nicotinamide adenine dinucleotide phosphate, reduced form
PCR	Polymerase chain reaction
RAGE	Receptor for AGEs
ROS	Reactive oxygen species
TUNEL	Terminal deoxynucleotidyl transferase dUTP nick end labeling
VSMC	Vascular smooth muscle cell
